# Meat-Inspection

**Published:** 1900-01

**Authors:** S. G. Burkholder

**Affiliations:** Chicago, ILL., Professor of Meat-Inspection in the M’killip Veterinary College


(To be continued.)

---

### MEAT-INSPECTION.

BY S. G. BURKHOLDER, M.D., V.S.,
CHICAGO, ILL.,
PROFESSOR OF MEAT-INSPECTION IN THE M'KILLIP VETERINARY COLLEGE.

MEAT-inspection is a part of veterinary medicine, the object of which is:

1. To discriminate unwholesome, innutritious, diseased meats from healthy, nutritious, and innocuous meats.

2. To prevent the substitution of carcasses or parts of carcasses of animals not usually used for human food, such as the horse, dog, cat, etc., for the carcasses of cattle, sheep, rabbits, etc.

3. To prevent the carcasses or parts of carcasses or other products of animals that have suffered from some febrile or infectious disease, or are in an advanced stage of pregnancy, from being sold to the unsuspecting public as wholesome meat.

4. To prevent animals suffering from contagious and infectious diseases from mingling with healthy animals or travelling over territories occupied by healthy animals, and thus spread the infection.

Meat-inspection may be divided into a science and an art. The science of meat-inspection consists of:

1. A thorough knowledge of the anatomy and comparative anatomy of cattle, sheep, and swine, as well as of the horse, dog, and other animals sometimes substituted for the former.

2. A thorough knowledge of the nature, symptoms, and pathological changes caused by the various diseases in animals, as well as the significance of these abnormal conditions.

3. A thorough knowledge of the nature of the infectious diseases transmissible from one animal to another or from animals to man, and the mode or channel through which transmission takes place.

The art of meat-inspection is the application of this knowledge in practice, thus preventing the transmission of disease from animal to animal or animal to man.

When we consider the object and importance of meat-inspection we at once realize the responsibilities resting upon the men who have charge of this work. The essential qualifications of a meat-inspector may be summed up as follows :

1. He must have a thorough knowledge of the science and art of meat-inspection.

2. He must be alert and conscientious, always realizing that the destiny and welfare of the public in a measure rests upon his ability and good judgment.

3. He must be resolute, courageous, and unyielding to either threats or bribes.

The importance of these three qualifications and the lamentable fact that they are not possessed by all in the meat-inspection service tempts me to discuss them a little more.

Since the power of making appointments to the Federal Meat-inspection Department has been placed under the ban of civil service the standard of the required qualifications of applicants for positions in this service has been raised, and the result is that the work is more efficient to-day than it ever was at a relatively less cost to the government.

Perhaps the paramount qualification of a meat-inspector is a thorough knowledge of the science and art of meat-inspection.

In the first place, he must be able to recognize the characteristic color, odor, firmness, and anatomical structure of the carcasses of all animals used as food, as well as those animals not used as food, which, however, might be substituted for such animals. For example : Horse meat may be substituted for that of the ox ; the carcass of a dog for that of a lamb, etc. Not that the product of these animals would be directly injurious to the health of those consuming them, nevertheless it seems unnatural, and our gustatory sense must undergo a prolonged course of training before we can relish such delicacies.

Of greater importance is a thorough knowledge of diseases of animals, especially those transmissible from one animal to another, or from animals to man. The symptoms and lesions characterizing each disease should be thoroughly understood and recognized, so that proper disposition can be made of the carcass without unnecessarily exposing susceptible animals or man to the disease.

While this may be considered the paramount qualification of the meat-inspector, it is by no means the only requisite. Unless he is conscientious and has the best interest of the public at heart, rather than his monthly check, his services can be easily dispensed with. He must be prompt, watchful, and alert, and investigate every suspicious action of those who might, by eluding his vigil, place upon the market meat that is unwholesome or diseased. The mercenary instinct of man is so strong that for the gain of a few dollars he is tempted to sell to the unsuspecting public meat from animals that are suffering from some infectious disease, as tuberculosis, actinomycosis, etc.

Again, a meat-inspector may be thoroughly competent in knowledge, conscientious, and alert, but he may lack courage and resolution, and yield to the bribes and perhaps threats of those whose conscientious scruples are overshadowed by their avaricious tendencies. Meat-inspectors who lack either ability, conscientiousness, or courage are a source of great danger to the public, and yet I regret to say they exist and will continue to exist as long as political influence is a stronger factor than competence in obtaining and retaining the position of meat-inspector.

Through the operations of the Bureau of Animal Industry, organized in 1884, in the Department of Agriculture, millions of dollars have no doubt been saved each year to the stock-breeders of this country as well as the lives of a number of people.

As early as 1870 the Commissioner of Agriculture recognized the importance of organized and scientific action to control diseases of animals, especially contagious pleuro-pneumonia and Texas fever, which diseases were then prevalent, causing disastrous losses and threatening to rapidly decimate the bovine tribe.

The large majority of veterinarians at that time were ignorant, superstitious empiricists. The few men who had scientific knowledge could not cope with the diseases because they were not supported by the Federal authorities.

In 1877 Congress granted the first appropriation, amounting to $10,000, for the purpose of controlling or eradicating these rapidly spreading diseases.

Dr. D. E. Salmon, of Washington, D. C., was one of the early promoters of this work, and when in 1884 Congress directed the organization of the Bureau of Animal Industry he was appointed chief of the bureau.

The stock-raising industry at this time was in a deplorable state. Our animals had been prohibited entrance into most of the European countries, and the prevalence of contagious pleuro-pneumonia and Texas fever in cattle, hog-cholera in swine, and scab in sheep, seriously interfered with interstate traffic at home, and the stock-raisers were almost panic-stricken. Dr. Salmon went to work at once introducing measures necessary to stop the spread of diseases among animals, and conducted original investigations to obtain further knowledge regarding the nature of these diseases.

The duties of the new bureau were most pressing, and the obstacles with which they had to contend interfered materially with their progress. Perhaps the paramount duty of the bureau at this time was to check and attempt to eradicate contagious pleuro-pneumonia, the disease which was rapidly devastating the bovine tribe, the losses of which amounted to millions of dollars to the stock-raisers. It is a deplorable fact that influential men, even the Commissioner of Agriculture himself, under whom the work was to be conducted, doubted the nature of the disease.

It was not until 1887 that Congress appropriated money and gave authority to purchase and kill all sick and exposed animals. By thus vigorously attacking the disease it was at once under control, and by the early part of 1892 it was considered completely eradicated. Not a single case has been reported since.

The inspection of imported cattle is so thorough and systematic that we have little fear of a fresh invasion. The complete eradication of this fatal and widely disseminated disease in the short period of five years, and with the relatively small expenditure of $1,500,000, is considered a notable achievement, and devolves great credit upon the men who were members of the bureau at that time.

Texas fever is another disease that was playing havoc among the bovines, arousing the government's attention as early as 1868.

The disease existed and its peculiarities were recognized in the latter part of the eighteenth century. Organized action was, however, not taken until after the establishment of the Bureau of Animal Industry.

In 1889 the bureau inaugurated a series of systematic investigations, and by the end of 1890 the nature, cause, and mode of infection of the disease were well understood. The disease has since been held in check, and we hope for a complete eradication at an early date.

Hog-cholera in swine and sheep scab are two other diseases that aroused the attention of the government at an early date, owing to the extensive destruction of animal life and the rapidity with which it spread.

The Bureau of Animal Industry steadily grew from the time of its inception, in 1884, until 1891, when it was found necessary to reorganize it into divisions, so that the work might be better systematized.

In March, 1891, Congress directed the Secretary of Agriculture to make an ante-mortem inspection of all cattle, sheep, and swine killed for foreign and interstate trade, and also authorized him to make a post-mortem inspection at his discretion. In this way the government was enabled to certify to the wholesomeness of exported meats.

At the same time microscopic inspection was inaugurated of all hog products exported to countries demanding microscopic inspection. In this way the confidence of foreign countries in the wholesomeness of our beef and pork products was re-established. The result is that our export trade in animal products is enormous.

This is, however, not all the Bureau of Animal Industry has accomplished. Let us consider a few of the more important contagious diseases and see what has been done in the way of controlling and eradicating them.

First, let us take up hog-cholera. We are all more or less familiar with the disease, and know how exceedingly fatal it is after once it gets into a herd of swine. For several years the bureau has been experimenting with an antitoxic serum with encouraging results. The serum is prepared by inoculating cattle, horses, mules, etc., with filtered, sterile or live cultures of hog-cholera and swine-plague bacilli, respectively, or solutions of their products, including the cell contents, extracts, and secretions. The quantities inoculated at first are small, but are gradually increased until large amounts can be inoculated without bad results. This must be continued for a period of from six to eight months.

The serum obtained from these animals has been used in a number of cases, with the result that about 80 per cent. of animals in diseased herds were saved.

This antitoxin method of treating hog-cholera and swine-plague is as yet in its infancy, but the results are so encouraging that we expect it to be a boon to the hog raiser.

The antitoxin treatment, both preventive and curative, of anthrax and symptomatic anthrax, is probably so well known that it needs no further comment. The mortality of both diseases is considerably reduced by this method of treatment. The percentage of loss from blackleg in herds is said to have been reduced from 10 to 20 per cent. to less than 1 per cent..

Texas fever is another disease that has caused great loss to the stock-raisers, but since the establishment of the Texas fever line and the enforcement of strict quarantine regulations by the bureau, the disease is fairly well under control.

Since it has been proven that the cattle tick (Boophilis bovis) is the transmitting agent of the infection various methods have been tried to destroy the parasite. The most encouraging results have thus far been obtained by dipping the cattle infested with these ticks. Southern cattle may be literally covered with these parasites and yet not suffer from Texas fever, because they have been rendered immune; but if these same ticks, or their progeny rather, find their way upon our Northern cattle they almost invariably transmit the disease.

The fact is now well established that where there are no ticks there will be no Texas fever, unless transmitted by direct inoculation; therefore if the ticks harbored by Southern cattle are destroyed before they mingle with our Northern cattle they will not transmit the disease.

The next object was to discover the best means of ridding Southern cattle from these disease-transmitting parasites. Dipping seems to be the most rational method, and various dipping solutions were tried, with the result that many of the solutions used were almost as destructive to the cattle as to the ticks. Thus far a saturated solution of sulphur in dynamo oil has given the best results. It seems reliable as far as destroying the ticks is concerned, but it is rather severe on the cattle dipped, so that a milder yet equally effective dipping solution would be preferable.

Thus, then, we have a method by which we can bring Southern cattle to our Northern pastures and stockyards with impunity; but the same method will not make it safe to take Northern cattle to Southern pastures until all the ticks south of the Texas fever line are killed, a rather stupendous undertaking.

Since Southern cattle, although literally covered with ticks, remain apparently well while our native cattle exposed to the same parasite usually become afflicted, we know that an immunity can be and is established. Many Southern cattle harbor the Texas fever organisms in their blood and yet show no outward symptoms of disease. If blood from these animals is inoculated into our native cattle they develop the symptoms of Texas fever. This proves not only that there is an immunity established in Southern cattle, but that the neutralizing agent or antitoxin is suspended in the blood of these animals. Why should not the blood-serum taken from Southern cattle and deprived of its active Texas fever organism give us an antitoxin which will, when inoculated into our native cattle, in graduated and repeated quantities, establish complete immunity in the animal treated without transmitting the disease itself? Experiments have been made along this line with encouraging results, and we have good reasons to suppose that some similar method will be found successful and put into practice at an early date.

Sheep-scab is another malady that has been a detriment to stock-raisers for years. The nature of the disease was not positively known until the early part of the present century. Since it is known that it is a parasitic disease, vigorous war has been waged against the mite causing the trouble. The best results thus far have been obtained from the use of dips. It is difficult, however, to prepare a dip that is destructive to the parasites and not injurious to the wool; the tobacco-and-sulphur and lime-and-sulphur dips have probably proven to be the most efficient thus far.

